# The diagnostic impact of fractional exhaled nitric oxide for asthmatic cough in nontuberculous mycobacterial pulmonary disease

**DOI:** 10.1186/s12890-024-03028-3

**Published:** 2024-04-29

**Authors:** Mari Miki, Keisuke Miki, Eri Akiba, Hiroyuki Kagawa, Yohei Oshitani, Takanori Matsuki, Kazuyuki Tsujino, Seigo Kitada, Ryoji Maekura, Hiroshi Kida

**Affiliations:** 1 Department of Respiratory Medicine, National Hospital Organization Osaka Toneyama Medical Center, Osaka, Japan; 2 Department of Internal Medicine, Tokushima Prefecture Naruto Hospital, 32 Kotani, Kurosaki, Muya-cho, Naruto-shi, Tokushima, 772-8503 Japan; 3 Senri Rehabilitation Hospital, Osaka, Japan; 4 Kodama Hospital, Hyogo, Japan; 5 Kitada Respiratory Clinic, Osaka, Japan

**Keywords:** Nontuberculous mycobacteria, Asthma, Exhaled nitric oxide, High-resolution computed tomography, Bronchial ectasia

## Abstract

**Background:**

Measurement of exhaled nitric oxide (FeNO) is a potentially useful diagnostic test for asthma. However, no study has explored the relationship between FeNO and respiratory symptoms of nontuberculous mycobacterial pulmonary disease (NTM-PD) complicated with asthma. The objective of this study was to assess the utility of measuring FeNO levels in patients with NTM-PD complicated by asthma.

**Methods:**

In this single-center retrospective cohort study, 140 NTM-PD patients with FeNO measured were enrolled. We selected NTM-PD patients who complicated with asthma as the NTM+BA group, defined using the following criteria: NTM patients with symptoms consistent with asthma, and NTM patients with symptomatic improvement after diagnostic therapy with ICS ± a long-acting beta 2-agonist (LABA). We then calculated a diagnostic cutoff point to distinguish between the NTM+BA groups and the NTM groups (all others). High-resolution computed tomography (HRCT) images were evaluated using the CT scoring system and their association with FeNO was examined.

**Results:**

A total of 89 patients were included in the study. (31 in the NTM+BA group and 58 in the NTM group). Compared with the NTM group, the NTM+BA group had higher rates of allergic disease (51.6% vs. 22.4%; *p*=0.0085) and higher FeNO values (median, 23 [interquartile range {IQR}, 15.0-43.0] ppb vs. median, 17 [IQR, 11.8-23.0] ppb; *p*=0.015). With diagnostic asthma care using mainly ICS/LABA with reference to the FeNO, most patients (91.0%, 20/22) in the NTM-preceding subgroup in the NTM+BA group demonstrated a prompt improvement of their symptoms and AFB culture findings did not worsen (Culture positive rate (%): Pre-treatment: 59.1% vs. Post-treatment: 40.9%, *p*=0.3660) at 6 months after starting diagnostic therapy. The optimal diagnostic cutoff point of FeNO to distinguish between the two groups was calculated as 21.5 ppb by the ROC curve (sensitivity 75%, specificity 71.93%, *p*<0.0001; area under the curve: 0.7989). No significant correlation was observed between FeNO and the severity of CT images in the patients.

**Conclusions:**

A certain number of patients with NTM-PD showed exacerbated respiratory symptoms due to asthmatic complications. Elevated FeNO levels suggest asthma complications, even in patients with NTM.

**Supplementary Information:**

The online version contains supplementary material available at 10.1186/s12890-024-03028-3.

## Background

Nontuberculous mycobacterial pulmonary disease (NTM-PD) cases are steadily increasing worldwide, with an annual prevalence of 3.2–9.8 per 100,000 people in North America [1, 2] and 14.7 per 100,000 person-years in Japan in 2014, approximately 2.6 of the incidence rate reported in 2007 [3]. This disease causes chronic cough, sputum, hemoptysis, fatigue, malaise, and weight loss [4] and reduces the quality of life in advanced stages.

Bronchial asthma (BA) is a common chronic airway inflammatory disease affecting 1%-29% of the population [5] and a common cause of chronic cough [6]. It is defined by a history of characteristic respiratory symptoms, such as wheezing, shortness of breath, chest tightness, cough, and evidence of variable expiratory airflow limitation [GINA 2023 [5]. However, the diagnosis of asthmatic cough is sometimes difficult because there are no validated tests or approved diagnostic criteria for asthma [7].

Many asthmatics have inflammation driven by type 2 cytokines [8], and increased fractional exhaled nitric oxide (FeNO) release from bronchial epithelium [9]. FeNO is modestly correlated with airway eosinophilic inflammation [10] and airway hyperresponsive [11], making it a useful marker for diagnosing asthma [12];-therefore, several diagnostic guidelines including FeNO have been proposed [13, 14]. These guidelines have established FeNO cutoff values and advocate a process whereby if asthma symptoms are present and the FeNO value is above the cutoff value, the patient should be diagnosed with asthma and begin treatment, including an inhaled corticosteroid (ICS). FeNO has also proven to be a useful marker for diagnosing asthmatic cough, showing a relatively high specificity of 0.85 in predicting adult asthma with chronic cough [15].

Although it is easy to speculate that the asthmatic component may be hidden in the coughing of patients with mycobacterial infections, asthmatic cough may be overlooked because NTM-PD itself causes a chronic cough. In addition, while ICS is an essential treatment for BA, there is some concern that this approach may lead to exacerbation of NTM-PD [16]. Conversely, ICS might have a positive impact on asthma symptoms in NTM and may lead to improved quality of life in patients.

The aim of the present study was to examine the diagnostic impact of measuring FeNO in predicting asthmatic cough (BA and cough variant asthma) in patients with NTM-PD, and the effects of ICS on NTM-PD complicated by asthma.

## Methods

### Study design and patients

This retrospective study was performed at the National Hospital Organization, Osaka Toneyama Medical Center (Osaka, Japan), a referral center for respiratory diseases. We enrolled patients with NTM-PD with FeNO measured because of a cough suspected of having asthma between April 1, 2014, and March 30, 2019, and for whom follow-up data were available for at least six months after the first FeNO measurement. (for details regarding the inclusion criteria and exclusion criteria, see the online supplemental methods).

This study was conducted in accordance with the principles of the Declaration of Helsinki and the experimental protocol for data involving human followed the Ethical Guidelines of the Japan Ministries of Health and Labour for Medical and Health Research Involving Human Subjects. The protocol was approved by the Clinical Research Review Board of the National Hospital Organization Osaka Toneyama Medical Center (approval number: TNH-A-2021026), and the need to obtain written informed consent was waived owing to the retrospective nature of the study.

### Data collection

Baseline clinical parameters, including age, sex, body mass index (BMI), smoking status, allergic disease history (allergic rhinitis, pollinosis, infantile asthma, and atopic dermatitis), and other comorbidities, were collected from each patient’s medical records at the first FeNO measurement. Data on the total eosinophil count, serum immunoglobulin E (IgE), anti-glycopeptidolipid-core IgA antibody, FeNO, spirometry, chest radiography, and high-resolution computed tomography (HRCT) findings were also collected. FeNO was measured using a NO analyzer (NIOX MINO or NIOX VERO; Aerocrine, Solna, Sweden).

### Definition of the NTM+BA and NTM groups

Asthma was defined by a history of variable respiratory symptoms, such as wheezing, shortness of breath, cough, and reversible expiratory airflow limitation, according to the Global Initiative for Asthma (GINA) [17] . However, airway reversibility can be difficult to prove in some asthmatics [18]. Therefore, we applied the following criteria to define NTM with asthmatic components (the NTM + BA group).

1) NTM patients with symptoms consistent with asthma, and

2) NTM patients with symptomatic improvement after diagnostic therapy with ICS ± a long-acting beta2-agonist (LABA).

It was preferred to the above conditions as the diagnosis of asthma that reversible FEV1, defined as an increase in the FEV1 of ≥12% and ≥200 mL after 2 inhalations of salbutamol, demonstrated an increase in the FEV1 of ≥200 mL after diagnostic therapy with ICS±LABA. Diagnostic ICS treatment for NTM-PD with a history of variable cough was performed, considering any findings suggestive of asthma, such as increased eosinophilia, an FeNO value ≥22 [19], atopic disposition, and a positive bronchial challenge test with methacholine. The remaining patients were assigned to the non-tuberculous mycobacterial (NTM) group. The grouping was performed by two chest physicians with more than 10 years of clinical experience.

### Radiological evaluations

HRCT was performed using a 64-row multidetector row CT scanner (SOMATOM Definition AS+; Siemens, Munich, Germany). Images at the first FeNO measurement ± 3 months were independently analyzed by two chest physicians, in the direction of a radiologist. We used the CT scoring system proposed by Lee et al. to assess HRCT findings of NTM-PD (Supplemental Table 1) [20].

**Table 1:** Baseline characteristics of NTM-PD patients

**Characteristics**	**Total (*n*=89)**	**NTM+BA vs. NTM**		
		**NTM+BA group (*n*=31)**	**NTM group (*n*=58)**	***P* value**
Age (years)	70.0 (60.5-76.0) 67.03333	69.0 (54.0-79.0)	70.0 (61.0-75.0)	0.9897
Sex, female, n (%)	83 (93.3%)	28 (90.3%)	55 (94.8%)	0.4220
Body mass index (kg/m^2^)	19.6 (17.8-21.8)	19.6 (18.4-21.2)	19.6 (17.4-22.0)	0.9083
onset age of NTM (y)	61.0 (52.5-69.0)	61.0 (52.0-74.0)	61.5 (52.8-68.0)	0.9656
Duration (y) of NTM	5.7 (1.0-11.5)	4.8 (0.7-11.7)	5.9 (1.6-11.4)	0.4641
Duration (y) of asthma	-	0.67 (0.0-5.9)	-	-
Smoking status (Never/Ex) n	79/10	28/3	51/7	>0.9999
Underlying diseases, n (%)				
Diabetes mellitus	6	1	5	0.6605
Collagen disease	4	2	2	0.6082
immunosuppressive agent use	2	1	1	>0.9999
Allergic disease, n (%)	29 (32.6%)	16 (51.6%)	13 (22.4%)	**0.0085**
FeNO (ppb)	18.0 (12.0-28.0)	23.0 (15.0-43.0)	17.0 (11.8-23.0)	**0.0151**
Blood eosinophil count (cells/μl)	140.0 (90.0-227.5)	160.0 (90.0-270.0)	130.0 (80.0-205.0)	0.1361
IgE (IU/ml)	60.0 (16.0-160.5)	58.5 (16.0-228.0)	60.0 (16.0-130.5)	0.4046
NB form	87 (97.8%)	29 (93.5%)	58 (100%)	0.1187
Cavity	23 (25.8%)	5 (16.1%)	18 (31.3%)	0.2031
AFB smear, positive	23 (25.8%)	10 (32.3%)	13 (22.4%)	0.3224
Anti-GPL core IgA antibody levels (U/ml)	2.50 (0.84-10.22)	1.35 (0.76-5.43)	4.20 (0.84-12.04)	0.1613
Mycobacterium species				
*M. avium*	54 (60.7%)	19 (61.3%)	35 (60.3%)	>0.9999
*M. intracellulare*	22 (24.7%)	8 (25.8%)	14 (24.1%)	>0.9999
*M. avium & intracellulare*	3 (3.3%)	1 (3.2%)	2 (3.5%)	>0.9999
*M. abscessus complex*	6 (6.7%)	2 (3.3%)	4 (8.4%)	>0.9999
*M. intracellulare+chelonae*	2 (2.2%)	1 (3.2)	1 (1.7%)	>0.9999
*M. gordonae*	2 (2.2%)	0 (0.0%)	2 (3.4%)	0.5409
Treatment of mycobacterium				
No treatment	37 (41.6%)	15 (48.4%)	22 (37.9%)	0.3733
On treatment	52 (58.4%)	16 (51.6%)	36 (62.1%)	
EM or CAM monotherapy monotherapy	21(23.6%)	6 (19.4%)	15 (25.9%)	0.6042
CAM -included regimen ≧2	5 (5.6%)	1 (3.2%)	4 (6.9%)	0.6543
CAM -included regimen ≧3	25 (28.1%)	9 (29.0%)	16 (27.6%)	>0.9999
Other regimen	1 (1.1%)	0 (0.0%)	1 (1.7%)	>0.9999
Duration from start any treatment of mycobacterium (y)	3.80 (1.40- 7.55)	2.05 (1.08-3.60)	3.80 (1.40-7.55)	0.0988
Pulmonary function test*				
FEV_1_, L	1.98 (1.71-2.25)	2.00 (1.67-2.35)	1.85 (1.76-2.25)	0.3812
FEV_1_, % predicted	85.2 (66.1-100.0)	87.3 (66.5-103.2)	82.7 (66.0-98.7)	0.7156
FEV_1_/FVC, %	79.2 (76.9-80.5)	79.2 (75.7-80.8)	78.8 (77.2-80.4)	0.8932
FEF50, L/s	1.56 (1.05-2.26)	1.71 (1.04-2.47)	1.49 (1.02-1.92)	0.4743
FEF25, L/s	0.40 (0.20-0.64)	0.47 (0.26-0.98)	0.36 (0.19-0.63)	0.3947
FEF50 % predicted	54.8 (35.6-80.3)	60.9 (35.5-84.6)	52.1 (35.2-76.3)	0.5205
FEF25 % predicted	34.1 (21.4-53.6)	35.6 (23.9-70.6)	33.7(19.3-53.6)	0.3947
**Asthma treatment**				
ICS	11 (12,4%)	11 (35.5%)	0 (0.0%)	**<0.0001**
Dose of ICS (μg/day)^**†**^ (FP Conversion amountdose)	0 (0-0)	0 (0-320)	0 (0-0)	**<0.0001**
OCS	1 (1.1%)	1 (3.2%)	0 (0%)	0.3483
LAMA	2 (2.3 %)	0 (0.0%)	2 (3.5%)	>0.9999
LABA	17 (19.1 %)	13 (41.9%)	4 (6.9%)	**0.0001**
leukotriene modifier, n (%)	9 (10.1 %)	9 (29.0%)	0 (0.0%)	**<0.0001**
theophylline, n (%)	2 (2.3%)	2 (6.5%)	0 (0.0%)	0.1187

Data represent the median (interquartile range) or n (%). *p*-values between the NTM + BA and NTM groups are shown

Pulmonary function test*: Total (*n*=78), NTM+BA group (*n*=31), NTM group (*n*=47)

^**†**^Fluticasone propionate equivalent. NTM-PD, nontuberculous mycobacterial pulmonary disease; *FeNO* Fractional exhaled nitric oxide, *IgE* Immunoglobulin E, *NB* Nodular bronchiectasis, *AFB* Acid-fast bacilli, *FEV*_*1*_ Forced expiratory volume in one second, *FVC* Forced expiratory capacity, *FEF50* Forced expiratory flow at 50% of forced vital capacity, *FEF25* Forced expiratory flow at 25% of forced vital capacity, *ICS* Inhaled corticosteroids, *OCS* Oral corticosteroids, *LAMA* Long-acting muscarinic antagonist, *LABA* Long-acting beta-agonists

### Statistical analyses

All statistical analyses were performed using the GraphPad Prism ver. 9 (GraphPad Software, San Diego, CA, USA) and JMP version 11 (SAS Institute, Cary, NC, USA). Continuous variables are expressed as median and interquartile range (IQR), and categorical data are expressed as numbers and percentages. Patient groups were compared using the Mann–Whitney U test for continuous variables and the χ^2^ test or Fisher’s exact probability test for categorical variables. The results among more than two groups were compared using the Kruskal-Wallis test followed by the Wilcoxon test. Statistical significance was set at *P* <0.05. Several patients had missing pulmonary function test and CT at the first FeNO measurement, in which case the number of patients was indicated in the tables and figures, and the missing values were omitted from the analysis. The sample size was estimated according to the previous report related to the FeNO cutoff values for the diagnosis of asthma [19], and the minimum number of patients was estimated to be 14 for each group using EZR (version 1.64, Saitama Medical Center, Jichi Medical University, Saitama, Japan).

## Results

### Baseline characteristics of the NTM+BA groups and NTM

**Fig. 1:**
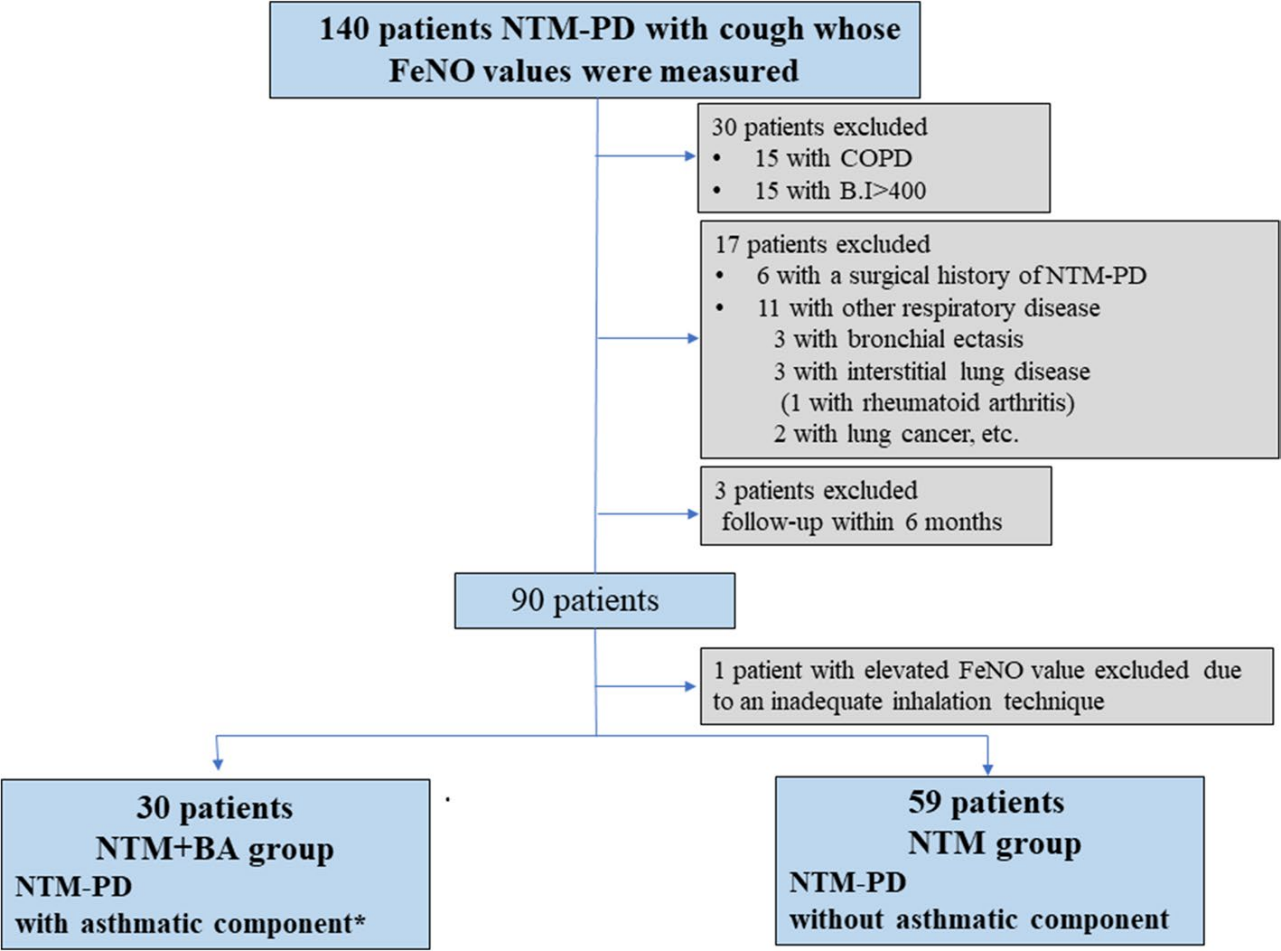
Study design. Patients were included if they were ≥20 years old, met the American Thoracic Society/Infectious Diseases Society of America criteria for nontuberculous mycobacterial pulmonary disease (NTM-PD) and measured fractional exhaled nitric oxide (FeNO) because of a cough and they were therefore suspected of having asthma. The patients who have any other respiratory disease were excluded. The patients were divided into the NTM+BA group, the patients with asthmatic component, and the NTM group (all others). B.I: Brinkman Index, ICS: inhaled corticosteroids. Asthmatic component*: Patients with clinical asthmatic symptoms and symptomatic improvement with diagnostic treatment with ICS. An FeNO value of ≥ 22 was considered for ICS use

A total of 140 patients with physician-diagnosed NTM-PD with cough and valid FeNO measurements were identified from medical records during the study period. Fifty-one patients were excluded, and the remaining 89 patients were enrolled in this study (Fig. 1).

Patients were divided into those with NTM complicated by asthma (NTM+BA group) and those without (NTM group), as described in the Methods section. The clinical characteristics of the patients are summarized in table 1. The median age at the onset of NTM was 61.0 (IQR, 52.5–69.0) years, female patients accounted for 90.3%, and the median duration of NTM was 5.7 (IQR, 1.0–11.5) years in all study participants. The NTM+BA group had asthma for up to 36.4 years, but the median duration was 0.67 (IQR,　0.0-5.9) years, and many patients had a short time since the onset of asthma. There was no significant difference in the prevalence of underlying diseases, such as diabetes mellitus or collagen diseases, between the two groups. In comparison to the NTM group, the NTM+BA group tended to more frequently show a history of allergic diseases, such as pollinosis, allergic rhinitis (22.4% vs. 51.6%; *p*=0.0085), and a higher FeNO value (23 [IQR, 15.0-43.0] ppb vs. 17.0 [IQR, 11.8-23.0] ppb; *p*=0.0151).

The majority of patients in both groups had nodularbrochiectasis (NB) according to CT, with fewer cavities than those previously reported in our hospital; however, Mycobacterium avium was most frequently cultured, consistent with our previous reports [21]. There were no significant differences in treatment regimens of NTM between the groups, but there was a significantly higher proportion of single-drug regimens, e.g. erythromycin or clarithromycin (CAM), and non-standard regimens than in our previous paper described above, where 72.0% of patients received standard treatments involved ≥3 drugs, including CAM [21]. The NTM group showed a higher median anti-GPL core IgA antibody level (4.20 [0.84-12.04]) than the NTM+BA group (1.35 [0.76-5.43]); however, the difference was not statistically significant.

In pulmonary function tests, FEV1% of predicted <50% was found in a few patients in both groups (NTM group:3/59 vs. NTM+BA group:1/30); however, the median FEV1/FVC ratio was 79.2%, and no patients had obstructive disorders. The evaluation of the peripheral airway function was considered using FEF50 and FEF25. In both groups, the FEF25 % predicted was low values (NTM+BA group: 35.6% [23.6-70.6] vs. NTM group: 33.7% [19.3-53.6%]), but no significant differences were observed between the two groups. At the first FeNO measurement, 35.5% (11/31) of the patients in the NTM+BA group were receiving ICSs because of a previous asthma diagnosis or symptoms suggestive of asthma, such as coughing and wheezing.

### Clinical characteristics of NTM+BA cases

As shown in table 2, the NTM+BA group included two types of patients: the NTM-preceding subgroup, which included patients in whom NTM onset preceded BA onset (*n*=19) or NTM and BA were diagnosed simultaneously (*n*=3), and the BA-preceding subgroup, which included patients in whom BA onset preceded NTM onset (*n*=9). The patients in the NTM-preceding subgroup were older than those in the BA-preceding subgroup (median age:72.0 [IQR, 62.8-79.0] years vs. 53.0 [IQR, 42.0-71.5] years; *p*=0.0379), however, the median age of NTM onset did not differ significantly between the subgroups in this study. In the NTM-preceding subgroup, NTM was followed by asthma onset at a median of 3.29 (IQR, 0.35-9.29) years later, the median duration of asthma was 0.21 (IQR, 0.00-3.75) years at the measurement of FeNO. In contrast, the BA preceding group had an average asthma duration of 7.67 years. Allergic diseases were found at high rates in both the NTM- and BA-preceding subgroups (45.4% vs. 66.7%) and peripheral eosinophil counts, FeNO values, and IgE levels were not significantly different between the subgroups.

**Table 2:** Clinical characteristics of NTM+BA patients

**Characteristic**	**NTM preceding subgroup (*n*=22)**	**BA preceding subgroup (*n*=9)**	***P* value**
Age (years)	72.0 (62.8-79.0)	53.0 (42.0-71.5)	**0.0379**
Sex, female, n (%)	20 (90.9%)	8 (88.9%)	1.0000
Body mass index (kg/m^2^)	20.1 (18.7-21.2)	18.6 (18.9-21.5))	0.5239
onset age of NTM (y)	63.5 (54.8-74.0)	53.0 (38.0-69.5)	0.1569
onset age of BA (y)	70.5 (62.5-77.0)	47.0 (15.5-65.5)	**0.0108**
Duration (years) between the onset of NTM and BA	3.29 (0.35-9.29)	7.58 (0.33-25.25)	0.5137
Duration (y) of asthma	0.21 (0.00- 3.75)	7.67 (0.67- 34.2)	**0.0021**
Allergic disease, n (%)	10 (45.4%)	6 (66.7%)	0.4331
FeNO (ppb)	23.5 (15.0-44.0)	22.0 (13.0-111.0)	0.9306
Blood eosinophil count (cells/μl)	155.0 (97.5-275.0)	200.0 (70.0-270.0)	0.9133
IgE (IU/ml)	57.0 (16.0-113.8)	161.0 (28.5-726.5)	0.1858
Anti-GPL core IgA antibody levels (U/ml)	1.690 (1.123-6.580)	1.270 (0.320-3.270)	0.2861
**Pulmonary function test**			
FEV_1_, L	1.92 (1.64-2.21)	2.21 (1.95-2.50)	0.0884
FEV_1_% of predicted (%)	90.9 (66.6-104.2)	79.3 (58.7-100.2)	0.6358
FEV_1_/FVC ratio (%)	78.0 (75.1-79.8)	81.2 (77.5-84.3)	0.0709
FEF50, L/s	1.80 (0.89-2.47)	1.29 (1.02-3.09)	0.8419
FEF25, L/s	0.47 (0.17-0.98)	0.42 (0.29-1.23)	0.8979
FEF50 % predicted	64.9 (48.1-83.1)	36.5 (32.9-91.0)	0.7431
FEF25 % predicted	34.7 (22.5-68.6)	36.4 (23.2-76.2)	>0.9999

Data represent the median (interquartile range) or n (%). p-values between the NTM-preceding subgroup and BA-preceding subgroup are shown. *NTM* Nontuberculous mycobacterial pulmonary disease, *BA* Bronchial asthma, *FeNO* Fractional exhaled nitric oxide, *IgE* Immunoglobulin E, *FEV*_*1*_ forced expiratory volume in one second, *FVC* Forced expiratory capacity, *FEF50* Forced expiratory flow at 50% of forced vital capacity, *FEF25* Forced expiratory flow at 25% of forced vital capacity

### Clinical course of NTM+BA cases treated with ICS/LABA

The clinical course of the NTM+BA group treated with ICS/LABA, while referencing the FeNO values, was analyzed. (table 3). The NTM-preceding subgroup of the NTM+BA group received a median of 320 μg/day (IQR:170-400 μg/day, expressed as fluticasone propionate equivalent) after FeNO measurement, and most patients (20/22) showed prompt symptom improvement. Two patients who had a wet cough presented due to NTM and showed improvement in cough frequency, but their wet cough persisted. Six months after the start of asthma therapy, AFB staining and culture findings did not worsen (Culture positive rate (%): Pre-treatment: 54.8% vs. Post-treatment: 38.7%, *p*=0.3087) in the NTM+BA group. In four cases in the NTM-preceding subgroup, culture conversion was confirmed after ICS therapy was initiated.

**Table 3:** Clinical course of NTM+BA patients treated with ICS/LABA

**Characteristics**	**NTM Preceding subgroup(*n*=22)**			**BA preceding subgroup (*n*=9)**		
	Pre-treat,	Post-treat,	*P* value	Pre-treat.	Post-treat.	*P* value
Use of ICS,n(%)	6 (27.3)	22(100.0)	**<0.0001**	5(55.5)	8(88.8)	0.2941
Dose of ICS (μg/day)	0(0-0)	320(170-400)	**<0.0001**	160(0-400)	450(320-800)	0.1533
Use of LABA, n (%)	7(31.8)	19(86.3)	**0.0027**	6(66.7)	7(77.7)	>0.9999
Use of LAMA, n (%)	0(0.0)	0(0.0)	>0.9999	0(0.0)	0(0.0)	>0.9999
Use of leukotriene modifier, n (%)	5(22.7’)	9(40.9)	0.3319	4(44.4)	6(66.7)	0.6372
Use of theophylline, n (%)	2(9.1)	2(9.1)	>0.9999	0(0.0)	1(11.1)	>0.9999
AFB stain positive, n (%)	8(36.4)	6(27.3)	0.7470	2(22.2)	2(22.2)	>0.9999
Culture positive, n (%)	13(59.1)	9(36.4)	0.3660	4(44.4)	3(44.4)	>0.9999

Data are presented as n (%) or median (interquartile range). *NTM* Nontuberculous mycobacterial pulmonary disease, *BA* Bronchial asthma, *ICS* Inhaled corticosteroids, *LABA* Long-acting β2-agonist, *LAMA* Long-acting muscarinic antagonist, *Pre-treat* Pre-treatment and *Post-treat* Post treatment, *AFB* Acid-fast bacilli, *p* values between Pre-treatment and Post-treatment are shown

### Case presentation: a case of NTM+BA in which ICS/LABA resulted in the improvement of NTM-PD

As noted above, four patients in the NTM+BA group showed improvement in NTM-PD after ICS therapy. Herein, we describe a representative case in which ICS/LABA inhalation resulted in the improvement of NTM-PD (Supplementary Figure 1). The patient had a history of pollen allergies and had developed difficult-to-treat pulmonary disease with M. avium. However, after high FeNO (69 ppb) was observed, she was diagnosed with refractory asthma complicated Aspergillus sensitization, and her AFB culture was changed to negative for 18 months with high dose of ICS/LABA.

### Optimal FeNO level for differentiating NTM with asthma from NTM without asthma

**Fig. 2:**
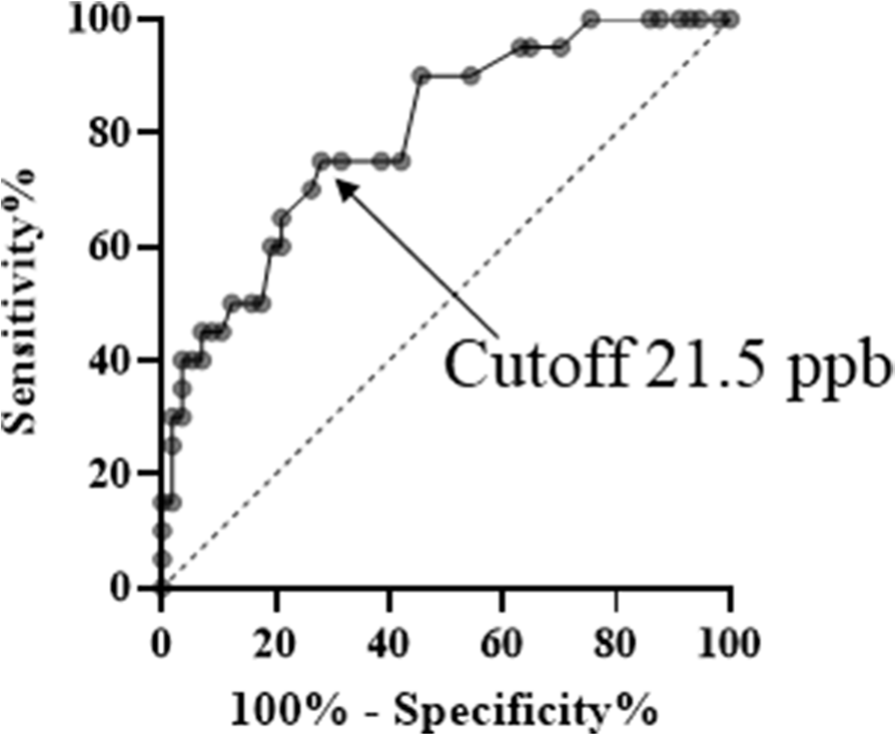
The optimal cutoff value for FeNO used to differentiate NTM cases accompanied by asthma from NTM-only cases. The values were calculated by the receiver operating characteristic (ROC) curve

We examined the FeNO value that differentiates the NTM+BA group from the NTM group, as defined in this study. It was calculated by using the receiver operating characteristic (ROC) curve, that the optimal diagnostic cutoff value for FeNO between the two groups was 21.5 ppb (sensitivity 75%, specificity 71.93%; *p*<0.0001), and the area under the curve was 0.7987 (Fig. 2).

### The relationship between the HRCT image features of NTM-PD and FeNO

**Fig. 3:**
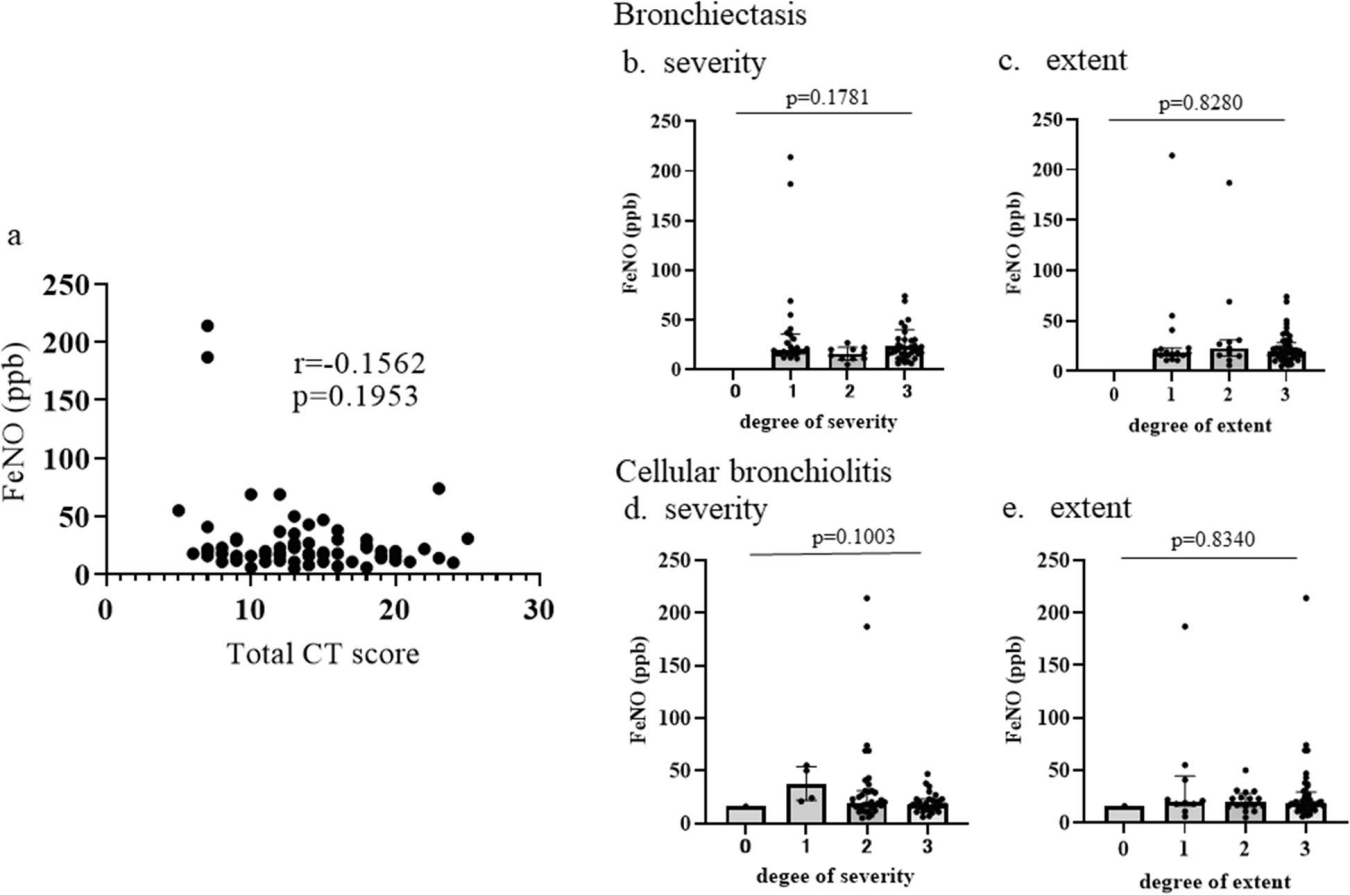
Relationship between the CT score from each image and FeNO. A total of 71 patients were evaluated for HRCT using a CT scoring system and were examined the relationship between FeNO and the scores, (NTM group [n-52] and NTM+BA group [*n*=19, cases without inhaled corticosteroids]). a shows FeNO and a total CT score. b.and c show FeNO and each CT score of bronchiectasis and d.and e show FeNO and each CT score of bronchiolitis. The CT scoreing system was shown in Supplemental Table 1

To further investigate the relationship between the imaging features of patients with NTM-PD and FeNO, the HRCT findings were examined. For this investigation, nine patients with no HRCT at FeNO measurement in total patients and nine patients in the NTM+BA group using ICS were excluded. A total of 71 patients were evaluated for HRCT using a CT scoring system consisting of five elements with four levels (0-3) (Supplementary Table 1) [20] (Supplementary Table 2). The total CT score (maximum 30) indicated that the image severity of NTM-PD was not correlated with FeNO (*r*=-0.1562, *p*=0.1953) (Fig. 3a). Furthermore, there were no significant differences in the severity or extent of bronchiectasis and bronchiolitis and FeNO (Fig. 3b, c and d, e). A positive correlation was noted between the total CT score and NTM duration (*r*=0.5340, *p* <0.00019) in this study (Supplementary Figure 2), indicating that the total CT score is an appropriate indicator of NTM-PD development. Using this image scoring system, the results of the image analysis of HRCT in our two study were as follows: the total score was markedly lower in the NTM+BA group than in the NTM group (average: 11.2 [95% confidence interval {CI} 8.787-13.53] vs. 14.4 [95% CI 13.13-15.67]; *p*=0.0061). Furthermore, the NTM+BA group showed milder bronchiectasis and cellular bronchiolitis than the NTM group (mean score: 1.6 [95% CI, 1.144-2.014] vs. 2.3 [95% CI, 2.088-2.565]; *p*=0.0034; and 2.1 [95% CI 1.800-2.305] vs. 2.4 [95% CI, 2.256-2.629]; *p*=0.0372, respectively) (Supplemental Table 2).

## Discussion

The NTM+BA group, which included NTM cases with asthmatic components, more frequently had an allergic history and had higher FeNO values than the NTM group, and the FeNO cutoff value for discriminating these groups was 21.5 ppb according to the ROC curve. The NTM-preceding subgroup treated with ICS+LABA after a tentative asthma diagnosis showed prompt improvement in coughing and no exacerbation of AFB culture within six months in the majority of cases. In addition, several patients showed culture conversion after initiation of ICS therapy.

Some asthma cases do not show clear airway reversibility [5], and several guidelines have proposed the use of FeNO to guide diagnosis [13, 14]. The FeNO cut-off value for differentiating between NTM and asthma-associated NTM was calculated as 21.5 ppb in this study, which is close to 22 ppb, the value used to differentiate between asthmatic and normal Japanese patients [19]. Furthermore, some NTM+BA patients were worsened their coughing after a common cold, pneumonia with fever, or hay fever attack. These findings agree with the frequently reported issue of asthmatic cough exacerbation triggered by viral infections [22] or hay fever [23], and indicate that the process of diagnosing and treating asthma complicated by NTM does not differ from that of usual asthma cases. Furthermore, the present study showed no marked difference in pulmonary function test findings between the NTM+BA and NTM groups, including in peripheral airway-related indices (FEF50, FEF25), indicating the usefulness of FeNO measurement in addition to the usual pulmonary function tests for asthma associated with NTM-PD as well as the usual diagnosis of asthma, as described above. However, there have been few detailed reports on coughing in NTM-PD, so further investigations into the mechanism of coughing are warranted.

The safety of ICSs in patients with NTM remains unclear. Bak et al. reported using a mouse model that comorbid allergic asthma exacerbated *M. avium* pulmonary infection (Mav-PI) by reducing the mycobacterium-specific Th17 response, which plays an important role in defense against intracellular pathogens [24]. They further showed that the aggravation of Mav-PI with a suppressed Th17 immune response was more prominent when allergic asthma was induced after *M. avium* infection than when asthma preceded it, and the comorbid allergic asthma in Mav-PI showed reduced disease progression over time, accompanied by a diminished degree of goblet cell hyperplasia with reduced IL-13 production. Crosstalk between Th17 and Th2 has also been shown to be mutually exclusive in patients with chronic rhinosinusitis with nasal polyps [25] .

In the present study, we report cases in which asthma symptoms improved without exacerbation of NTM-PD for six months after ICS therapy was started or intensified. Given the present findings and previous reports of Th2-Th17 crosstalk over several months, suppression of Th2 inflammation by ICS may lead to the improvement of clinical symptoms without exacerbation of NTM-PD. Based on the findings of this study, the NTM-PD patients with atopic disposition and variable symptoms or triggering symptoms suggestive of asthma should be aggressively examined for airway reversibility and FeNO to determine whether or not they have asthma complications. If possible, bronchial challenge testing with methacholine should be performed before starting ICS treatment to strengthen the asthma diagnosis. In this study, only four patients in the NTM+BA group underwent bronchial challenge testing with methacholine and showed a positive reaction. In this small number of cases, we found, surprisingly, that ICS-based asthma treatment improved in several cases of refractory NTM-PD itself. There is a major question of how the ICS dose should be determined, but a short-term response to treatment with an ICS dose similar to that of usual asthma treatment can be considered for patients with NTM-PD who have positive findings suggestive of asthma. However, prolonged long-term ICS administration might exacerbate NTM-PD, as previously reported, and it may be necessary during ICS treatment to assess Th2 inflammation and reduce the ICS dose appropriately over time, with discontinuation of ICSs considered when asthma symptoms improve sufficiently. However, in some cases, continued ICS administration can be necessary due to increased Th2 inflammation. Further study is needed to determine the appropriate duration and dose of ICS therapy for asthma-complicated NTM-PD.

In this study, we also showed that the FeNO value was not correlated with the severity of total CT images or the degree of bronchiectasis or cellular bronchiolitis in NTM-PD patients. Bronchiectasis and bronchiolitis progress with the development of NTM-PD [20]. In the present study, both bronchiectasis and cellular bronchiolitis were more severe in the NTM group than in the NTM+BA group, but FeNO values were lower in the NTM group. Regarding bronchiectasis, it was reported that bronchiectasis patients with *Pseudomonas aeruginosa* infection had significantly lower FeNO values than those without *Pseudomonas* infection, and that there was no correlation between FeNO and the number of bronchiectatic lung lobes [26]. Our results were consistent with this report, suggesting that high FeNO values in NTM patients were indicative of asthma, regardless of the degree of imaging findings.

### Limitations

The present study has several limitations. First, this study had a retrospective design and was a single-center study with a relatively small sample size. Admittedly, the NTM and NTM+BA groups had slightly different backgrounds, including smoking history. However, the major statistical backgrounds (e.g. age, sex, and severity of bacteriology of NTM) were consistent in both groups, Second, the diagnosis of the NTM+BA group was a clinical diagnosis, and the possibility cannot be ruled out that other bronchiolitis responsive to ICS/LABA may be included in the group. Bronchial challenge testing with methacholine should be performed for the more accurate diagnosis of asthma. Third, we were unable to identify a clear indicator that could distinguish pure NTM from NTM with asthmatic complications. Fourth, the majority of NTM-PD patients with asthma complications in this study had been asthmatic for only a few months at the measurement of FeNO. Larger and longer studies of multiple centers are needed to confirm the results of this study, and further investigations are required to identify patients indicated for ICS therapy and optimal dosage of ICS for NTM complicated by asthma in order to prevent the development of NTM.

## Conclusions

In conclusion, asthma and NTM are chronic inflammatory diseases with different mechanisms, and their clinical courses depend on their treatment. Our study showed, for the first time, that FeNO is useful for diagnosing NTM-PD complicated by asthma, as it is for diagnosing typical asthma. We hope that further studies will be conducted to elucidate the immune mechanisms underlying these complications and to identify biomarkers that can be used as therapeutic indices of asthma when ICS are used in NTM-PD.

### Supplementary Information

**Supplementary Material 1.** 

## Data Availability

The data collected and analyzed during the current study are available from the corresponding author on reasonable request.

## Abbreviations

BA: Bronchial asthma
FeNO: Fractional exhaled nitric oxide
NTM-PD: Nontuberculous mycobacterial pulmonary disease
FEV_1_: Forced expiratory volume in 1 s
ICS: Inhaled corticosteroids
LABA: Long-acting beta2-agonist
HRCT: High-resolution computed tomography
IQR: Interquartile range
AFB: Acid-fast bacilli
FVC: Forced vital capacity
BMI: Body mass index
IgE: Immunoglobulin E
NB: Nodularbronchiectasis
EB: Ethambutol
CAM: Clarithromycin
AMK: Amikacin
