# Supplementary material for: The diagnostic impact of fractional exhaled nitric oxide for asthmatic cough in nontuberculous mycobacterial pulmonary disease
Source: BMC Pulm Med. 2024 Apr 29;24:210. doi: 10.1186/s12890-024-03028-3 (PMC11059766; doi:10.1186/s12890-024-03028-3)
Supplement: Supplementary file 1 — Supplementary Material 1. [file 12890_2024_3028_MOESM1_ESM.docx]

**Online supplement**

**Title:**

**The diagnostic impact of fractional exhaled nitric ox1ide for asthmatic cough in nontuberculous mycobacterial pulmonary disease**

**Online Supplement Material**

1. **Supplemental methods**
2. **Supplementary tables 1-2**
3. **Supplementary figures 1-3**

**Supplemental methods**

*Study design and patients*

The inclusion criteria were as follows:

1. age ≥20 years.
2. meeting the American Thoracic Society/Infectious Diseases Society of America criteria for NTM-PD [1].
3. valid FeNO data.

The exclusion criteria were as follows:

(1) combination with other respiratory diseases, such as COPD showing a forced expiratory volume in 1 s (FEV1) to forced vital capacity (FVC) ratio of <70%, in accordance with the documents of the Global Initiative for Chronic Obstructive Lung Disease (GOLD) [2], bronchiectasis (including bronchial lesions associated with rheumatism), interstitial lung disease (ILD), sequelae of pulmonary tuberculosis, and lung cancer;

(2) a history of lung resection for NTM-PD, lung cancer, or other respiratory diseases.

(3) a smoking history of >10 pack-years; and (4) insufficient follow-up time (<6 months after the first FeNO measurement).

Supplemental Table 1. CT scoring system [3] .

| **CT Finding** | **Score** | | | |
| --- | --- | --- | --- | --- |
|  | **0** | **1** | **2** | **3** |
| Bronchiectasis (9 points) |  |  |  |  |
| Severity* | Absent | Mild | Moderate | Severe |
| Extent[^†^](javascript:popRef('tblfn2')) | Absent | 1–5 | 6–9 | >9 |
| Mucus plugging[^†^](javascript:popRef('tblfn2')) | Absent | 1–5 | 6–9 | >9 |
| Cellular bronchiolitis (6 points) |  |  |  |  |
| Severity[^‡^](javascript:popRef('tblfn3')) | Absent | Mild | Moderate | Severe |
| Extent[^†^](javascript:popRef('tblfn2')) | Absent | 1–5 | 6–9 | >9 |
| Cavity (9 points) |  |  |  |  |
| Diameter, cm | Absent | <3 | 3–5 | >5 |
| Wall thickness, mm | Absent | <1 | 1–5 | >5 |
| Extent, no. | Absent | 1–3 | 4–5 | >5 |
| Nodule extent[^†^](javascript:popRef('tblfn2')) (3 points) | Absent | 1–5 | 6–9 | >9 |
| Consolidation extent[^†^](javascript:popRef('tblfn2')) (3 points) | Absent | <3 | 3–5 | >5 |

Definition of abbreviation: CT = computed tomography.

Maximal possible score = 30 points.

*Mild = bronchus diameter greater than adjacent vessel diameter; moderate = bronchus diameter two to three times vessel diameter; severe = bronchus diameter greater than three times vessel diameter.

†Number of involved segments.

‡Mild = identifiable, peripheral lung, 1 cm from pleura; moderate = definite, involvement greater than 1–3 cm from pleura; severe = extensive, extending to central lung.

Supplemental Table 2. The comparison of HRCT image features between the NTM+BA and NTM groups.

| **Characteristic** | **Total (n=80)** | | **NTM+BA group**  **(including ICS) (n=28)** | | **NTM group (n=52)** | | **P value*** | **NTM+BA group**  **(ICS free)**  **(n=19)** | | **P value^†^** |
| --- | --- | --- | --- | --- | --- | --- | --- | --- | --- | --- |
|  | **Mean** | **95% CI** | **Mean** | **95% CI** | **Mean** | **95% CI** |  | **Mean** | **95% CI** |  |
| **Bronchiectasis** |  |  |  |  |  |  |  |  |  |  |
| Severity**^‡^**  (3 points) | 2.1 | 1.920-2.330 | 1.8 | 1.390-2.110 | 2.3 | 2.088-2.565 | **0.0170** | 1.6 | 1.144-  2.014 | **0.0034** |
| Extent**^§^**  (3 points) | 2.4 | 2.205-2.570 | 2.1 | 1.814-2.472 | 2.5 | 2.203-2.736 | 0.0668 | 2.2 | 1.725-  2.591 | 0.2336 |
| Mucus plugins**^§^**  (3 points) | 0.7 | 0.537-0.829 | 0.6 | 0.355-0.756 | 0.8 | 0.552-0.978 | 0.5011 | 0.6 | 0.3013-  0.8098 | 0.6591 |
| **Cellular bronchiolitis** |  |  |  |  |  |  |  |  |  |  |
| Severity**^\|\|^**  (3 points) | 2.3 | 2.180-2.470 | 2.1 | 1.887-2.327 | 2.4 | 2.256-2.629 | **0.0396** | 2.1 | 1.800-  2.305 | **0.0372** |
| Extent**^§^**  (3 points) | 2.4 | 2.221-2.579 | 2.3 | 1.923-2.577 | 2.5 | 2.264-2.698 | 0.3679 | 2.4 | 1.968-  2.769 | 0.7162 |
| **Cavity** |  |  |  |  |  |  |  |  |  |  |
| Diameter,cm  (3 points) | 0.4 | 0.220-0.555 | 0.4 | 0.004-0.711 | 0.4 | 0.219-0.589 | **0.0178** | 0.3 | -0.1401-  0.7717 | **0.0265** |
| Wallthickness  ,mm (3 points) | 0.6 | 0.329-0.771 | 0.4 | 0.014-0.843 | 0.6 | 0.350-0.881 | **0.0091** | 0.3 | -0.1401-  0.7717 | 0.0571 |
| Extent, no  (3 points) | 0.5 | 0.262-0.687 | 0.2 | 0.006-0.4546 | 0.6 | 0.313-0.918 | 0.1762 | 0.2 | -0.09341-  0.5145 | 0.0890 |
| **Nodule** |  |  |  |  |  |  |  |  |  |  |
| Extent^§^  (3 points) | 0.2 | 0.103-0.296 | 0.1 | -0.015-0.229 | 0.3 | 0.116-0.384 | 0.3653 | 0.1 | -0.05794-  0.1632 | 0.2248 |
| **Consolidation** |  |  |  |  |  |  |  |  |  |  |
| Extent§  (3 points) | 1.8 | 1.592-2.008 | 1.4 | 1.072-1.785 | 2.0 | 1.753-2.247 | **0.0397** | 1.6 | 1.144-  2.014 | 0.3544 |
| **Total** **CT score** (30 points) | 13.3 | 12.25-14.40 | 11.3 | 9.481-13.16 | 14.4 | 13.13-15.67 | **0.0057** | 11.2 | 8.784-  13.53 | **0.0061** |

The CT score (Supplemental table 1) was used to classify the CT severity between the two groups. NTM: nontuberculous mycobacterial pulmonary disease; BA: bronchial asthma; NTM+BA group (including ICS); NTM+BA group including cases with inhaled steroids; ~~ICS-free~~ NTM+BA group (ICS free); NTM+BA group not including the cases with inhaled steroids; The P value * between the NTM+BA (including ICS) and NTM groups and the P value † between the NTM and NTM+BA (ICS free) groups are shown. A χ2 test was used to compare the two groups.

‡0=absent, 1 = bronchus diameter greater than adjacent vessel diameter; 2 = bronchus diameter two–three times the vessel diameter; 3 = bronchus diameter greater than three times the vessel diameter.

**^§^**Number of involved segments.

The NTM group showed more severe bronchiectasis and cellular bronchiolitis than the NTM+BA group (mean score: 2.3 [95% confidence interval, 2.088-2.565] vs. 1.8 [95% CI, 1.390-2.110]; p=0.0170; and 2.256 [95% CI 2.256-2.629] vs. 2.1 [95% CI, 1.887-2.327]; p=0.0396, respectively). Regarding these characteristics, the extent of the diseased segment did not significantly differ between the two groups. In addition, in the NTM+BA group, bronchiectasis of the right middle lobe was more common than that of the left lingular segment, albeit without significance (Supplemental figure 2). Regarding cavities, significant differences in the cavity diameter and wall thickness were also found between the groups (p=0.0178 and p=0.0091, respectively). Even in the group without inhaled steroids, the results were largely consistent with the results between the two groups, except for the wall thickness of the cavity lesion.


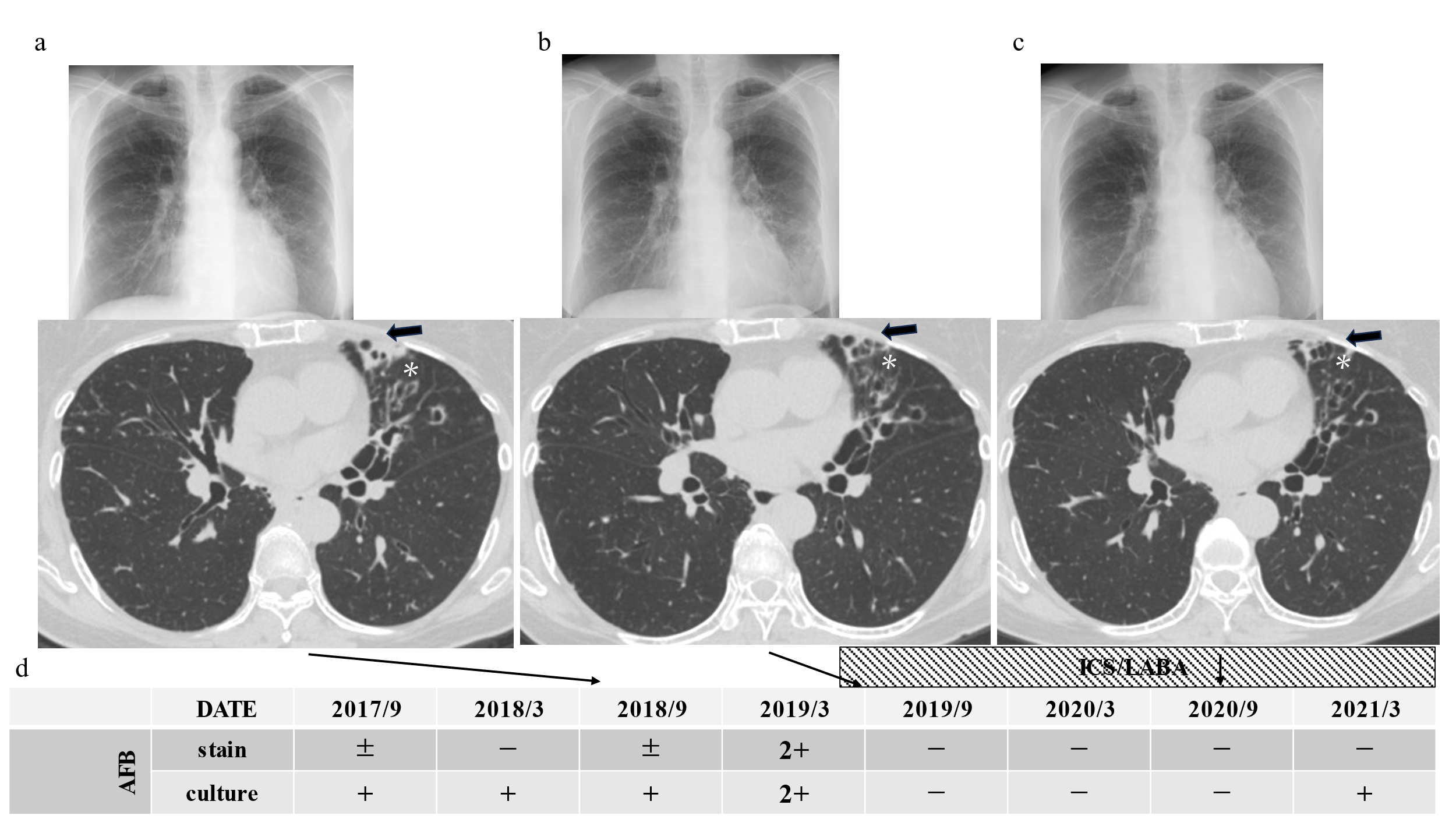


Supplemental Figure 1. Chest XPs and HRCT images of a case of NTM+BA in which ICS/LABA resulted in the improvement of pulmonary M. avium infection. a, before ICS/LABA therapy started. b, after 2 months of ICS/LABA therapy started. c, after 15 months of ICS/LABA therapy started. d. clinical course of acid-fast bacilli stain and culture. ICS/LABA therapy was started in July 2019.

A 67-year-old woman with a history of pollen allergies developed pulmonary disease with M. avium from 55 years of age (in 2007) despite initial treatment with rifampicin, ethambutol (EB), and clarithromycin (CAM), which failed to achieve culture conversion. She had received re-treatment with CAM+EB+moxifloxacin or sitafloxacin+amikacin (AMK) inhalation or tobramycin inhalation, three times in total, from 61 to 67 years of age (Supplementary figures 1a), but the culture had been continuously positive since 62 years at 67 years of age (in 2019) and experienced exacerbation triggered by the common cold during the hay fever season. She was treated with ceftriaxone and AMK injections for one week, and her fever improved, but the cough persisted. In July 2019, high FeNO (69 ppb) and eosinophilia (320/μl) were observed, and she was started on ICS/LABA for the diagnosis of BA with budesonide formoterol fumarate hydrate (BUD/FM; 320 μg/9 μg/day). However, at the end of August, she showed eosinophilia progression (940/μl), elevated IgE levels (1220 IU/ml with a positive reaction for Aspergillus-specific IgE [1.09 UA/ml]), and increased shadows in the right lower lobe on chest X-ray. The shadow improved quickly with sulbactam sodium and cefoperazone sodium (Supplementary figures 1b); however, her bronchoalveolar lavage fluid in early September revealed hypereosinophilia (10.3%). The BUD/FM dose was increased to 960 μg/27 μg/day as she was diagnosed with severe asthma with fungal sensitization. After ICS/LABA therapy was intensified, her respiratory symptoms improved and her AFB culture was negative for 18 months (Supplementary figures 1c). After 10 months, HRCT showed improvement in bronchial wall swelling and consolidation around the bronchus.

Total CT score

Duration of NTM (years)

r=0.5340

p=<0.0001

Supplemental Figure 2. Relationship between NTM duration and total CT scores.

A figure shows the relationship between the total CT scores and NTM duration in total of 71 patients of NTM-PD.


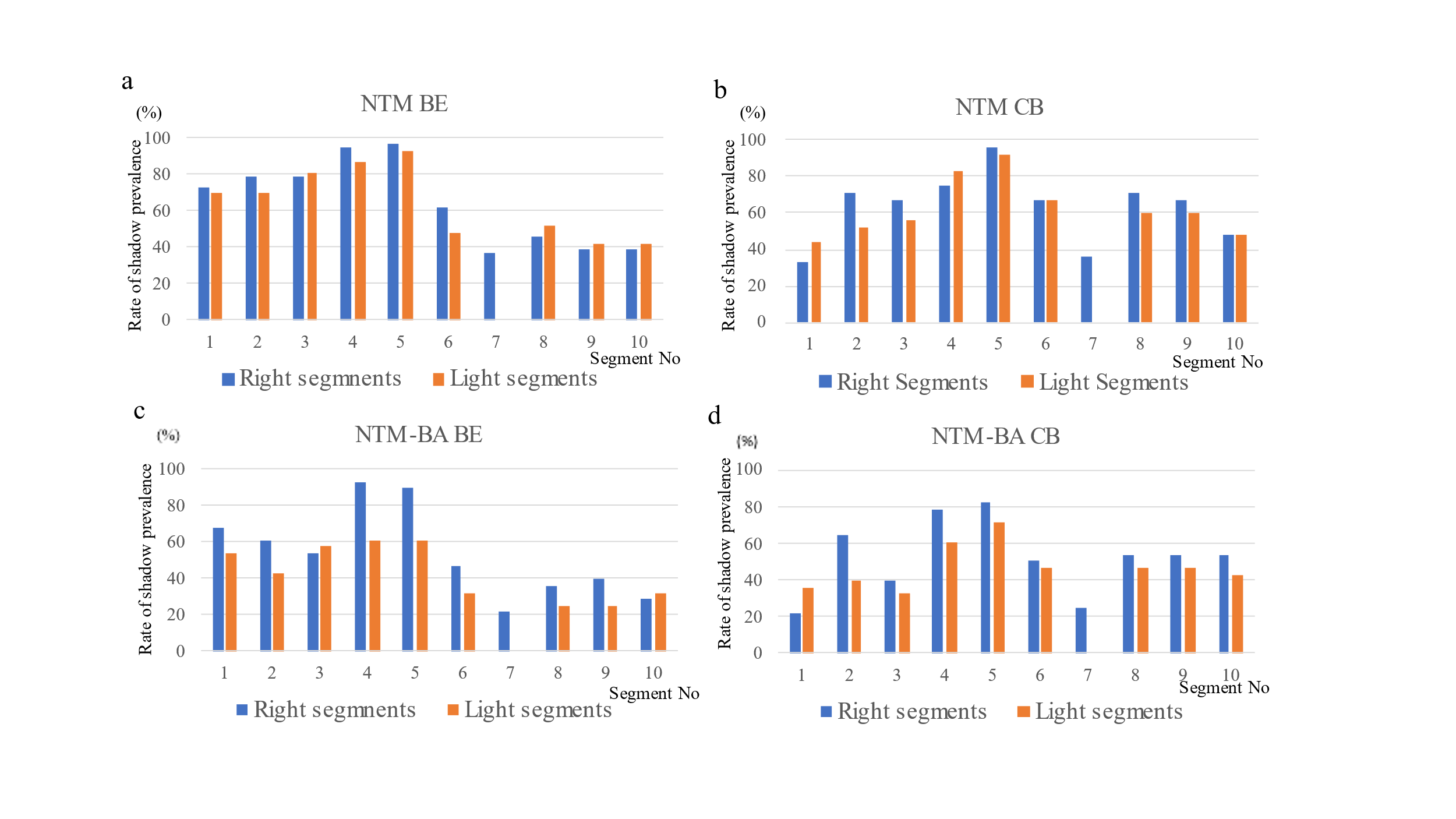
Supplemental Figure 3. Ratios of the involved segments in the NTM and NTM + BA groups.

1. and c. show bronchiectasis (BE). and b and d show cellular bronchitis (CB).

In the NTM+BA group, bronchiectasis of the right middle lobe was more common than that of the left lingular segment, albeit without significance.

References

1 Griffith DE, Aksamit T, Brown-Elliott BA*, et al.* An official ATS/IDSA statement: diagnosis, treatment, and prevention of nontuberculous mycobacterial diseases. *Am J Respir Crit Care Med* 2007; 175: 367-416.

2 Vogelmeier CF, Criner GJ, Martinez FJ*, et al.* Global Strategy for the Diagnosis, Management, and Prevention of Chronic Obstructive Lung Disease 2017 Report. GOLD Executive Summary. *Am J Respir Crit Care Med* 2017; 195: 557-582.

3 Lee G, Lee KS, Moon JW*, et al.* Nodular bronchiectatic Mycobacterium avium complex pulmonary disease. Natural course on serial computed tomographic scans. *Ann Am Thorac Soc* 2013; 10: 299-306.
